# Rhizosphere *Mortierella* strain of alfalfa exerted weed growth inhibition by inducing expression of plant hormone-related genes

**DOI:** 10.3389/fmicb.2024.1385992

**Published:** 2024-06-17

**Authors:** Taotao Du, Xudong Qu, Yibo Wang, Meixuan Li, Xihu Qie, Jing Jin, Yuxuan Gao, Zengyu Wang, Kejian Lin, Chao Yang, Juan Sun

**Affiliations:** ^1^Key Laboratory of National Forestry and Grassland Administration on Grassland Resources and Ecology in the Yellow River Delta, College of Grassland Science, Qingdao Agricultural University, Qingdao, Shandong, China; ^2^College of Plant Health and Medicine, Qingdao Agricultural University, Qingdao, Shandong, China; ^3^Institute of Grassland Research of CAAS, Hohhot, Inner Mongolia, China

**Keywords:** *Digitaria sanguinalis*, *Mortierella*, inhibition mechanism, plant hormone, *Mortierella* strain MXBP304

## Abstract

**Introduction:**

Weeds are significant factors that detrimentally affect crop health and hinder optimal herbage yield. Rhizosphere microorganisms play crucial roles in plant growth, development, and nutrient uptake. Therefore, research focusing on weed control through the lens of microorganisms has emerged as a prominent area of study. The oil-producing fungus *Mortierella*, which is known for its numerous agricultural benefits, has garnered significant attention in recent years.

**Methods:**

In this study, we conducted inoculation experiments in a controlled artificial culture climate chamber to investigate the effects of differential hormones and differentially expressed genes in the stems and leaves of *Digitaria sanguinalis* using Liquid Chromatography Tandem Mass Spectrometry and RNA-seq techniques, respectively. Additionally, Pearson’s correlation analysis was used to establish correlations between differential hormones and growth indicators of *Digitaria sanguinalis*.

**Results and discussion:**

The results demonstrated that inoculation with *Mortierella* sp. MXBP304 effectively suppressed aboveground biomass and plant height in *Digitaria sanguinalis*. Furthermore, there was significant upregulation and downregulation in the expression of genes involved in the synthesis and metabolism of phenylalanine and L-phenylalanine. Conversely, the expression of genes related to tryptophan, L-tryptophan, and indole was significantly downregulated. The addition of *Mortierella* sp. MXBP304 can influence the gene expression associated with phenylalanine and tryptophan synthesis and metabolism during *Digitaria sanguinalis* growth, subsequently reducing the relative contents of phenylalanine and tryptophan, thereby directly inhibiting *Digitaria sanguinalis* growth.

## 1 Introduction

Alfalfa (*Medicago sativa*) is not only a leguminous forage with the largest planting area but also a high-protein forage that is indispensable for the production of high-quality milk (Sun et al., 2013). However, in the production process, herbage is threatened by weeds, such as the main grain crops, and the harm to weeds often leads to planting failure or substantial yield reduction (Lin et al., 2013). Among them, the annual grass species *Digitaria sanguinalis* is both a malignant farmland weed and a dominant weed in alfalfa fields in summer and autumn (Shao and Wu, 1999; Zhang et al., 2013). Competition leads to reduced crop and forage yield and quality (Yang and Yang, 1998). Worldwide, chemical methods are still the dominant way to deal with weeds, despite their negative impacts. Chemical herbicides directly enter agricultural products with the harvest of nutrients, such as crops, endangering human health. Environmental pollution and ecological damage caused by herbicide abuse are becoming increasingly serious (Bagavathiannan et al., 2019). Hence, there is an urgent need for environmentally friendly weed control technologies. The adoption of effective methods to control weeds within a reasonable range and to minimize their impact on forage yield and quality, livestock production, and environmental safety has become a technical bottleneck for weed control in pasture fields (Qian, 2019).

Microorganisms play key roles in the competitive growth of plants. In recent years, the regulation of plant growth and soil nutrient availability by microorganisms has become a research hotspot in the international academic community (Attitalla et al., 2020). In the direction of microbial weed control, plant pathogenic microorganisms and soil rhizosphere microorganisms are usually used to control weeds, among which pathogenic microorganisms are mainly used to control weeds by leaf spraying. In contrast, soil rhizosphere microorganisms are used to manage indigenous microbial communities in the field to achieve ecological weed control (Qu and Feng, 2019). Some studies have found that soil microorganisms destroy the seed bank of soil weeds and inhibit their growth in the field through strategies such as attracting seeds by chemotaxis and producing enzymes and toxins to kill seeds (Kennedy, 2020). Non-parasitic soil microorganisms, which are common in the rhizosphere, can significantly inhibit weed growth and have great potential as weed microbial inhibitors (Malik and Charaya, 2013). Bacteria isolated from plant roots and soil can effectively inhibit weeds in lawns and wheat fields (Kennedy, 2016). The *Erwenella* S7, extracted from the roots of *Digitaria sanguinalis,* completely inhibits the seed germination of setaria (Dan et al., 2002). *Pseudomonas fluorescens* in soil can effectively inhibit the malignant invasive weed *Bromus tectorum* (DB), Germination of *Aegilops cylindrica* (JG), and *Taeniatherum caput-medusae* (MH). After 3 years of continuous use, DB, JG, and MH were reduced by 50%, while other pastures were not affected. This is precisely because of the selectivity of soil rhizosphere microorganisms, which can be used to manage weeds in cultivated land, pastures, and on both sides of roads (Kennedy, 2018). This study proposed a new strategy for the micro-ecological management of grassland weeds.

Soil microbial communities play an important role in the competition for growth between alfalfa and weeds during the alfalfa planting process. The abundance of *Mortierella*, *Alternaria,* and *Cladosporium* in the rhizosphere fungal community is significantly higher than that in the rhizosphere, with many weeds (Yang et al., 2022). *Ligustrisporites* and *Cladisporites* can easily cause plant diseases (Bensch et al., 2012). Non-pathogenic microorganisms of *Mortieresporites* can promote the growth and development of crops and have become an international research hotspot (Ozimek and Hanaka, 2020). *Mortierella elongata* plays a significant role in nutrient transformation and promotes plant growth in mineral soils (Li et al., 2018). Adding *Mortierella elongata* to orchard soils can significantly increase the content of available phosphorus, potassium, calcium, magnesium, and boron (Tamayo-Vélez Aosorio, 2018). In sand ginger black soil, *Mortierella* strains significantly increased the content of soil available nitrogen and phosphorus and the activities of β-glucosidase and phosphatase (Qiao et al., 2012). Inoculants of *Mortierella capiformis* increased rhizosphere bacterial diversity and significantly changed the composition of the rhizosphere bacterial community (Li et al., 2020). These studies showed that microbial *Mortierella* fungi have significant effects on crop growth and development, soil nutrient conversion, and microbial communities.

In the process of plant growth and development, in addition to the influence of microorganisms, inherent plant hormones are important factors for plant growth and development. When the growth environment of plants changes, the signaling pathway of plant hormones is activated, thus playing an important role in responding to various biological and abiotic stresses. Transcription factors play an important role in plant resistance to various environmental stresses (Wang et al., 2006). *Trichoderma* has been shown to promote root growth and development by upregulating the auxin synthesis pathway in maize (Xu et al., 2020). *Piribospora indica* can activate the glutathione-ascorbic acid cycle of plants, thereby improving the antioxidant cycle. The overall increase in crop yield (Cao et al., 2023). *Mortierella* isolates from maize rhizosphere soil also increase the concentrations of IAA and ABA in the roots (Li et al., 2020). *Sporoides* in the soil of saffrons planted in India increased the content of chlorophyll and carotenoids in plants (Wang H. W. et al., 2018). This means that there is a mutual relationship among microorganisms, plant hormones, and other pathways.

**Figure 1:**
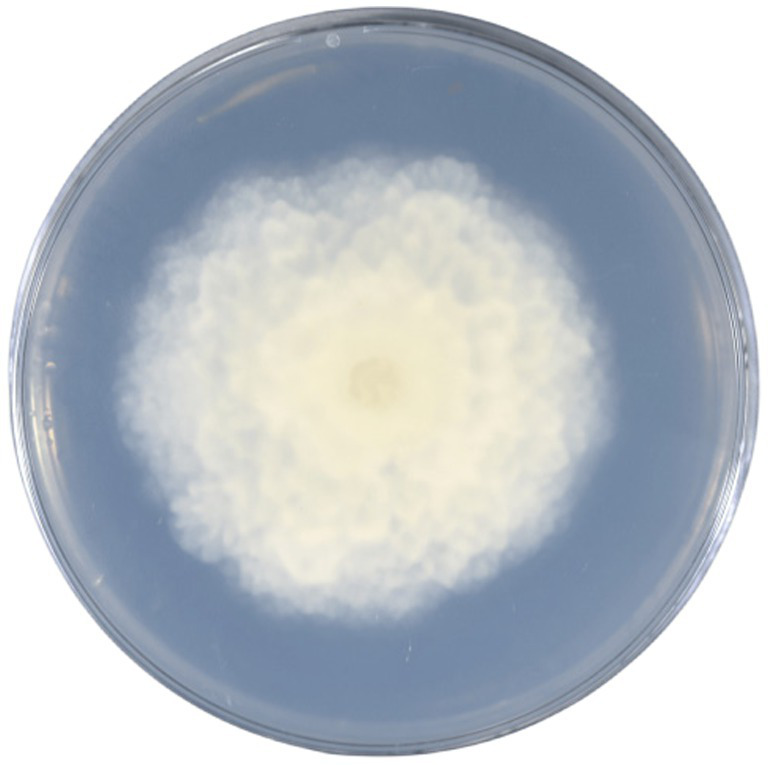
*Mortierella* strain MXBP304.

In the previous study, it was found that the abundance of *Mortierella* in the rhizosphere fungal community of alfalfa with fewer weeds was significantly higher than that of alfalfa with more weeds (Yang et al., 2022), so whether the occurrence of weeds was related to the abundance of *Mortierella* fungi. Therefore, in this study, *Mortierella* sp. MXBP304 (Figure 1) screened from alfalfa rhizosphere was used as the key strain to study whether the strain was related to weeds in alfalfa fields. We hypothesized that the *Mortierella* sp. MXBP304 plays a disproportionate role in soil nutrient cycling and weed growth. Therefore, we hypothesized that (1) inoculation of soil with the rare fungus *Mortierella* sp., MXBP304, may inhibit the plant height and biomass of *Digitaria sanguinalis*. (2) Inoculation with *Mortierella* sp. MXBP304 inhibits weed growth by coupling changes in gene expression with changes in plant hormones. Therefore, we conducted a pot experiment to clarify the inoculant-plant-native microbiome relationship. We studied the changes in plant hormones in inoculated plants treated with spores and controls, and detected differential gene expression in inoculated stems and leaves using RNA-seq.

## 2 Materials and methods

### 2.1 Isolation and identification of the rhizome of *Medicago sativa*

The study site is located in the Demonstration Park of Modern Agricultural Science and Technology of Qingdao Agricultural University, Qingdao, Shandong Province (36°26 ‘22”N, 120°04’ 43 “E, elevation 1 m). This area has a warm temperate monsoon climate, with an average annual temperature of 12.3°C and an average annual precipitation of about 680.5 mm, and an average annual air pressure of 1008.7 Mpa. The annual frost-free period is 251 days, and the annual sunshine duration is 2,708 h. The soil at the test site is loam.

Alfalfa root soil was collected at the test site and immediately brought to the laboratory to isolate the fungi. Fungal separation methods were based on the experimental instructions and separation method of *Pinellia* (Zhang et al., 2014). After grinding the collected rhizosphere soil, it was passed through a 60-mesh sieve for use. Samples (2 g) and 18 ml of sterile water were placed in a 50-ml conical bottle, and the samples were fully shaken to disperse evenly in the water. Then, 0.1-ml dispersion was absorbed on the PDA medium, according to the colony morphology on the plate, a different color was observed, and a single colony was selected for continuous purification. The purified strains were inoculated on a Petri dish, spread evenly, and cultured in an incubator at 30°C at a constant temperature. Colonies with growth rates and morphologies similar to the morph were screened, and DNA was cleaved from the lysate using the Cymbidium Direct Expansion Kit. ITS1F (5’-TTCCGTAGGTGAACCTGCGG-3’) and ITS4R (5’-CATATCAATAAGCGGAGGAA-3’) were used to amplify the 18SrDNA gene of the fungal genome. PCR amplification was performed in a 50-μl system, including 1 × T3 Mix 46 μl upper and downstream primers (10 μmol/L) (1 μl each) and DNA template (2 μl). The amplification procedure for the PCR reaction was as follows: pre-denaturation at 98°C for 3 min; 35 cycles of denaturation at 98°C for 10 s, annealed at 55°C for 15 s, extended at 72°C for 20 s; extension for 2 min at 72°C; and storage at 4°C. The PCR products were sequenced after they were gelled and recovered.

### 2.2 Pot experiment design and sample collection

For the pot experiment, we collected soil from alfalfa fields in Jiaozhou City, Shandong Province, China. Before planting, 1 kg of sterilized air-dried soil was filled in a pot, the inoculation method of fungal *Mortierella* was referred to in a previous study (Li et al., 2018). *Mortierella* was expanded and cultured for 7 days at 28°C/180 revolutions in the dark. The amplified strains were filtered through sterile gauze and washed several times with sterile distilled water. Finally, pure strains were suspended in sterile deionized water for soil inoculation. There were two experimental groups: treatment and control. The treatment group was inoculated with 200 ml of suspension (~5 g of fresh mycelia) of the morycetes strains, and 200 ml of sterile deionized water was added to the control group, with four replicates for each treatment. The seeds were transplanted after germination, and the corresponding bacterial suspensions and sterile water were applied twice within a week of transplantation. Sterile water was applied regularly. All pots were cultured in the same climate chamber with 16 h of light (28 ± 2°C) and 8 h of darkness (20 ± 2°C). Relative humidity was maintained at 60–80%.

The chlorophyll content (SPAD) of the second and third true leaves was determined using a portable chlorophyll analyzer, plant height was determined with a ruler, and fresh weight was measured using a balance. The stems and leaves of *Digitaria sanguinalis* were immediately frozen in liquid nitrogen and stored at −80°C for the determination of enzyme activity and plant hormones. Enzyme activity was determined using an enzyme activity kit provided by Jiangsu Coming Biological Company.

### 2.3 Determination of plant hormones in plant stems and leaves

Fresh plant samples were harvested, immediately frozen in liquid nitrogen, ground into powder (30 Hz, 1 min), and stored at −80°C until needed. Fifty milligrams of plant sample were weighed into a 2-ml plastic microtube, frozen in liquid nitrogen, and dissolved in 1 ml of methanol/water/formic acid (15:4:1, V/V/V). Ten microliters of mixed internal standard solution (100 ng/ml) were added to the extract as an internal standard (IS) for quantification. The mixture was vortexed for 10 min, centrifuged for 5 min (12,000 r/min, and 4°C), transferred to clean plastic microtubes, evaporated to dryness, dissolved in 100 μl 80% methanol (v/v), and filtered through a 0.22-μm membrane filter for further LC–MS/MS analysis (Cai et al., 2014; Floková et al., 2014; Li Y. et al., 2016).

The sample extracts were analyzed using UPLC-ESI-MS/MS (Mortierellacn/). The analytical conditions were as follows: LC column, Waters ACQUITY UPLC HSS T3 C18 (100 mm × 2.1 mm i.d., 1.8 μm); solvent system, water with 0.04% acetic acid (A), acetonitrile with 0.04% acetic acid (B); gradient program, started at 5% B (0–1 min), increased to 95% B (1–8 min), 95% B (8–9 min), finally ramped back to 5% B (9.1–12 min); flow rate, 0.35 ml/min; temperature, 40°C; injection volume: 2 μl (Niu et al., 2014; Cui et al., 2015; Xiao et al., 2018).

Linear ion trap (LIT) and triple quadrupole (QQQ) scans were acquired on a triple quadrupole-linear ion trap mass spectrometer (QTRAP), QTRAP® 6,500+ LC–MS/MS System, equipped with an ESI Turbo Ion-Spray interface, operating in both positive and negative ion mode and controlled by Analyst 1.6.3 software (Sciex). The ESI source operation parameters were as follows: ion source, ESI+/−; source temperature, 550°C; ion spray voltage (ISV), 5,500 V (positive), −4,500 V (negative); and curtain gas (CUR), 35 psi. Phytohormones were analyzed using scheduled multiple reaction monitoring (MRM). Data acquisition was performed using Analyst 1.6.3 software (Sciex). Multiquant 3.0.3 software (Sciex) was used to quantify all the metabolites. Mass spectrometry parameters, including the declustering potentials (DP) and collision energies (CE) for individual MRM transitions, were performed with further DP and CE optimization. A specific set of MRM transitions was monitored for each period according to the metabolites eluted within this period (Cui et al., 2015; Šimura et al., 2018).

Identified metabolites were annotated using the Kyoto Encyclopedia of Genes and Genomes (KEGG) compound database,^1^ and annotated metabolites were then mapped to the KEGG pathway database.^2^ Pathways with significantly regulated metabolites mapped were then fed into metabolite set enrichment analysis (MSEA), and hypergeometric test *p*-values determined their significance.

### 2.4 RNA-seq and analysis of plant stems and leaves

To identify genes associated with plant hormones, RNA concentration was measured using the Qubit® RNA Assay Kit in a Qubit®2.0 Fluorometer (Life Technologies, CA, USA). RNA degradation and contamination were monitored on 1% agarose gels, and RNA purity was checked using a NanoPhotometer® spectrophotometer (Implen, CA, USA). RNA integrity was assessed using the RNA Nano 6,000 Assay Kit on a Bioanalyzer 2,100 system (Agilent Technologies, CA, USA). Sequencing libraries were generated using the NEBNext® UltraTM RNALibrary Prep Kit for Illumina® (NEB, USA) following the manufacturer’s recommendations, and index codes were added to attribute sequences to each sample. Then, RNA sequencing (RNA-Seq) and data processing were performed using the Illumina HiSeq platform at Metware Biotechnology Co., Ltd. (Wuhan, China), according to Wang Y. et al. (2018).

Fastp was used to filter the original data, primarily to remove the reads with adapters. When the N content in any sequencing reads exceeded 10% of the base number of the reads, the paired reads were removed. When the number of low-quality (Q ≦ 20) bases contained in reads exceeded 50% of the bases of the reads, these paired reads were removed (Chen et al., 2018). All subsequent analyses were performed using clean reads. Transcriptome assembly was performed using Trinity software (Ning et al., 2017). For the study samples, DESeq2 was used to analyze the differential expression between the two groups (Love et al., 2014; Varet et al., 2016). The *p*-value was corrected using the Benjamini & Hochberg method. The corrected p-value and |log2foldchange| are used as the threshold for significant difference expression, and the pathway significant enrichment analysis was performed using the KEGG database.^3^

### 2.5 Joint analysis of the metabolome and transcriptome

The differential hormones and differential genes in this experiment were simultaneously injected into the KEGG pathway map to better understand the relationship between genes and plant hormones. At the same time, Pearson correlation analysis was also performed on microorganisms with significant abundance changes and soil indicators to understand the relationship between microorganisms and soil indicators. Plant hormones and plant growth indicators were also jointly analyzed.

### 2.6 Statistical analysis

Student’s *t*-test was performed using SPSS software (version 19.0) to assess the effects of *Mortierella* inoculation on soil and plant basic properties, and the diversity of rhizosphere bacterial and fungal communities. Pearson correlation analysis was performed using SPSS software (version 19.0) to evaluate the relationship between the abundance of rhizosphere microbial modules, the relationship between key elements and soil properties, and the relationship between plant hormones and genes. Significance at the 0.001, 0.01, and 0.05 levels is represented by ***, ****, and *, respectively. Origin2021 was used to visualize the results of the Pearson correlation analysis.

## 3 Results

### 3.1 Morphological and phylogenetic tree of strains

**Figure 2:**
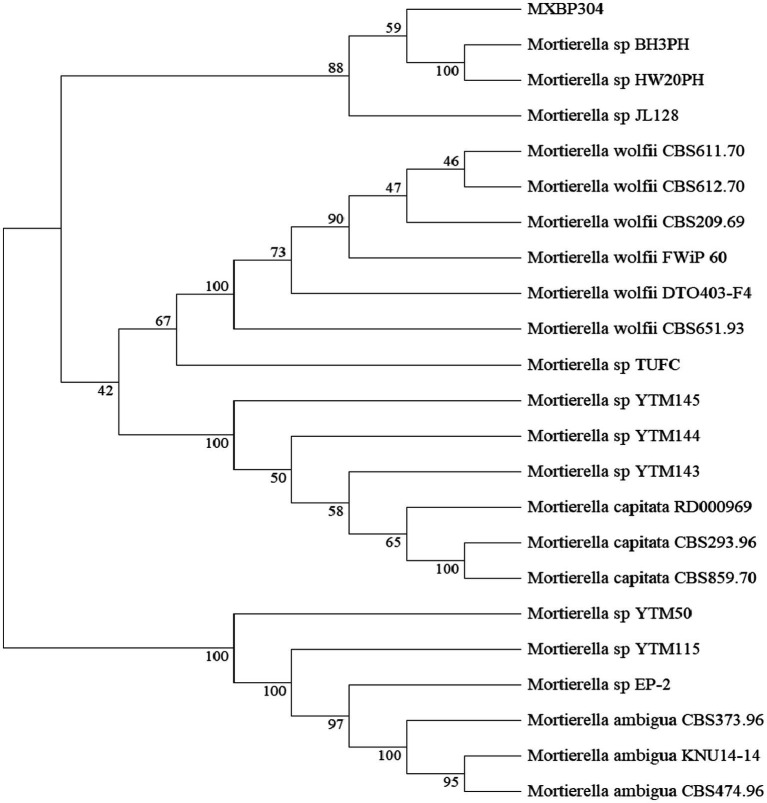
Phylogenetic identification of the isolated *Mortierella* strain (referred to as isolate MXBP304).

In the GY medium, the *Mortierella* strain showed growth of white cotton flocculent mycelia accompanied by garlic flavor (Figure 1). BLAST was used to search for the homologous gene of MXBP304, and a phylogenetic tree was constructed in MEGA11.0, using the neighbor-joining method. The results showed that the isolated strain (MXBP304) had the closest phylogenetic relationship with *Mortierella* and could be identified as Mortiella (Figure 2). However, it is not closely related to other *Mortierella* strains and may represent a new *Mortierella*. According to systematic nomenclature, the isolated strain BP-04 of *Mortierella* was named MXBP304, and the strain was stored in the typical Chinese culture preservation center with the storage number CCTCC NO:M20211660.

### 3.2 Effects of *Mortierella* sp. MXBP304 on basic plant parameters

**Figure 3:**
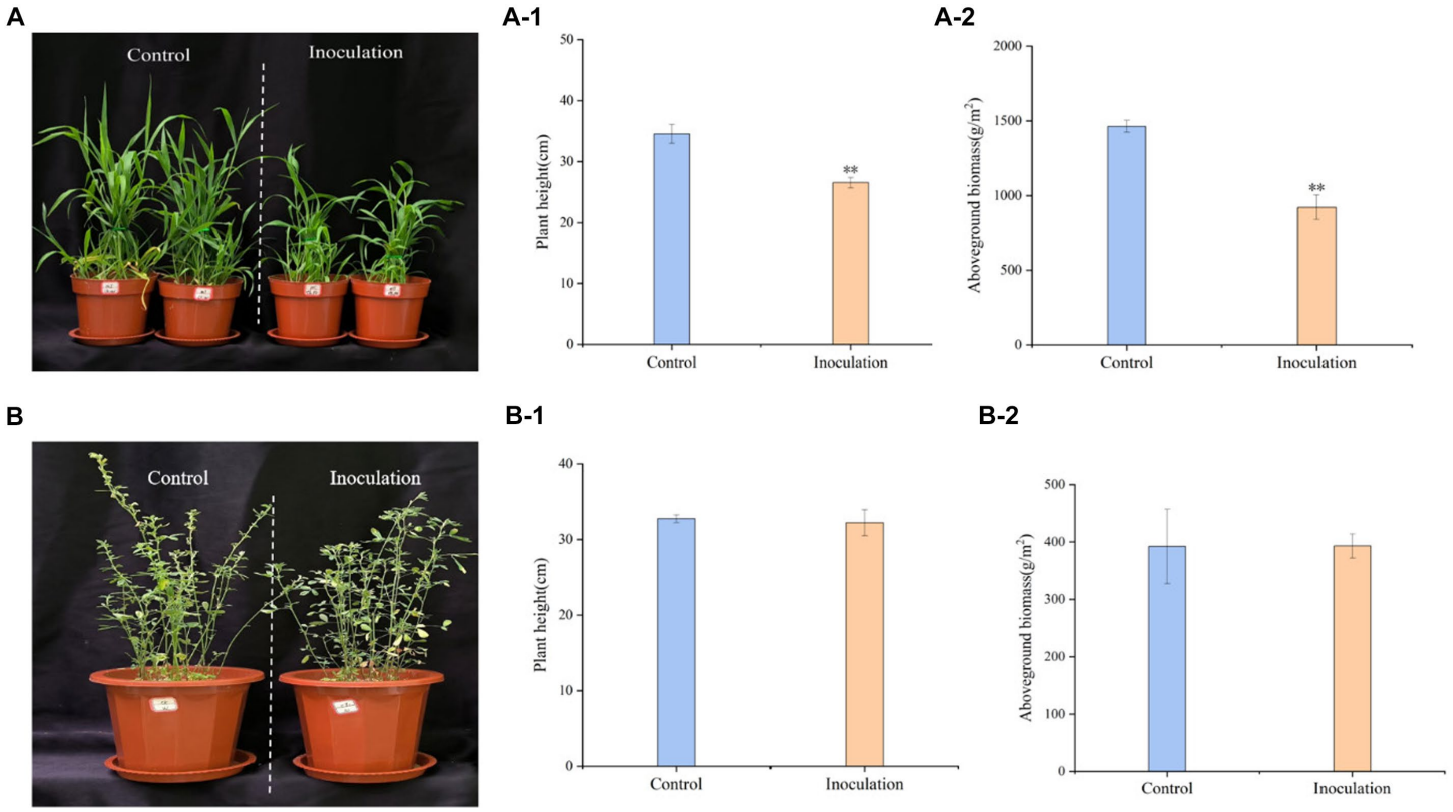
Phenotypic effects of *Mortierella* inoculation on *Digitaria sanguinalis* and alfalfa. **(A)** *Digitaria sanguinalis;* **(B)** alfalfa. **(A-1)** is the aboveground biomass of *Digitaria sanguinalis,* and **(A-2)** is the plant height of *Digitaria sanguinalis*. Panel **(B-1)** is the aboveground biomass of alfalfa and **(B-2)** is the plant height of alfalfa. *p < 0. 05, **p < 0. 01. *N* = 4.

Inoculation treatment significantly reduced the height of *Digitaria sanguinalis*, aboveground biomass, and chlorophyll content of *Digitaria sanguinalis* weeds, but had no significant effect on underground biomass (Figure 3A). At the same time, *Mortierella* had no significant effects on the biomass or plant height of the target forage alfalfa (Figure 3B).

### 3.3 Effects of inoculation of *Mortierella* sp. MXBP304 on the physiological indexes of *Digitaria sanguinalis*

**Figure 4:**
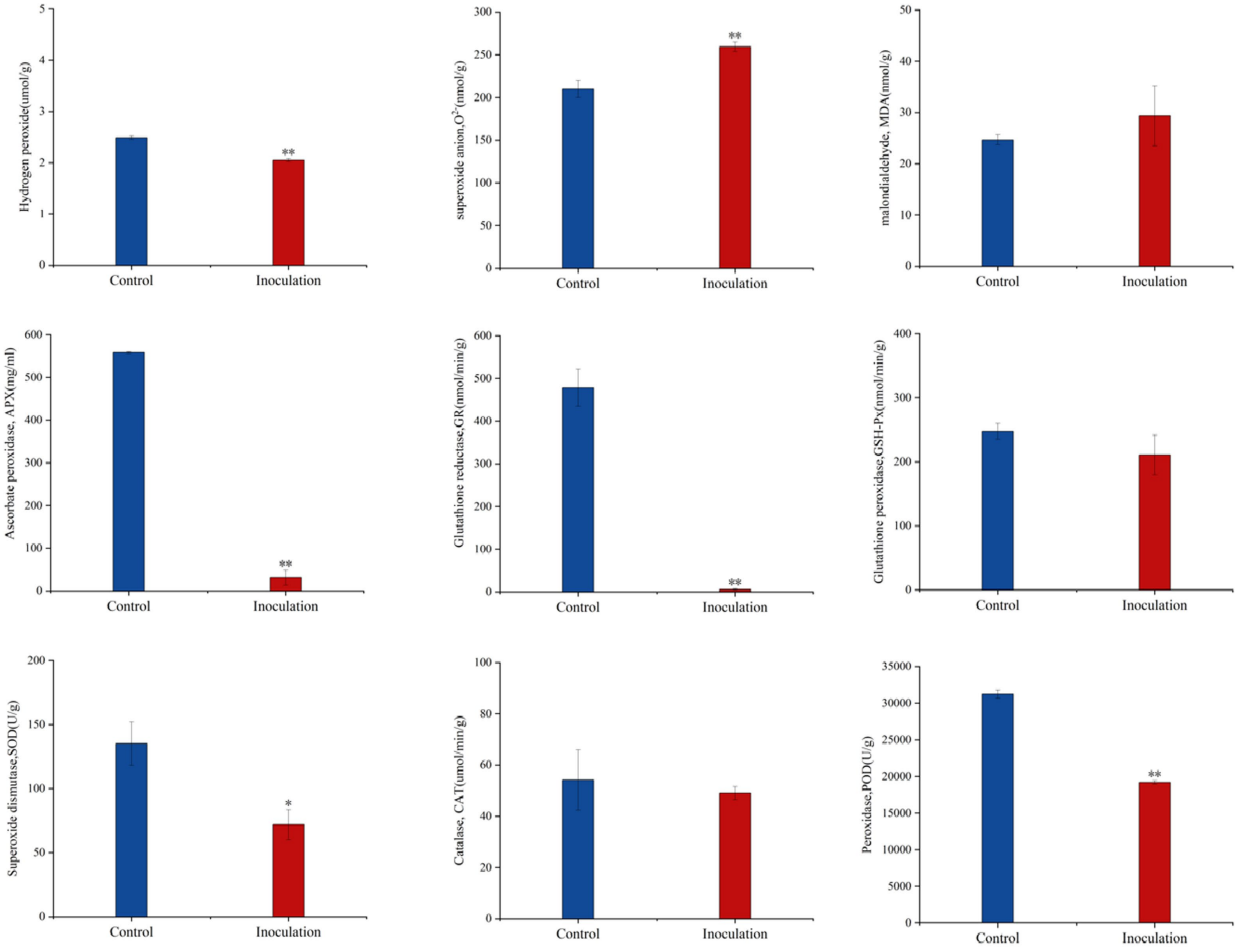
Effect of *Mortierella* sp. MXBP304 inoculation on physiological indexes of *Digitaria sanguinalis*. In the figure, the abscissa is the inoculated MXBP304 group and the uninoculated control group, and the ordinate is the enzyme activity. *p < 0.05, **p < 0.01. *N* = 3.

Compared with the control group, after inoculation with *Mortierella* sp. MXBP304, in stems and leaves, the content of superoxide anion (O^2−^) increased significantly, the content of hydrogen peroxide (H_2_O_2_) decreased significantly, and the content of malondialdehyde (MDA) did not change significantly. The significant increase in superoxide anion content causes an imbalance in free radicals in plants and has a negative impact on their growth and development. At the same time, the activity of the key protective enzymes in the leaves was measured, and it was found that inoculation significantly decreased the activity of ascorbate peroxidase (APX), glutathione reductase (GR), peroxidase (POD), and superoxide dismutase (SOD) in the leaves. There was no significant effect on catalase (CAT) or glutathione peroxidase (GSH-PX) enzyme activities (Figure 4).

### 3.4 Plant hormone content in stems and leaves of *Digitaria sanguinalis*

**Figure 5:**
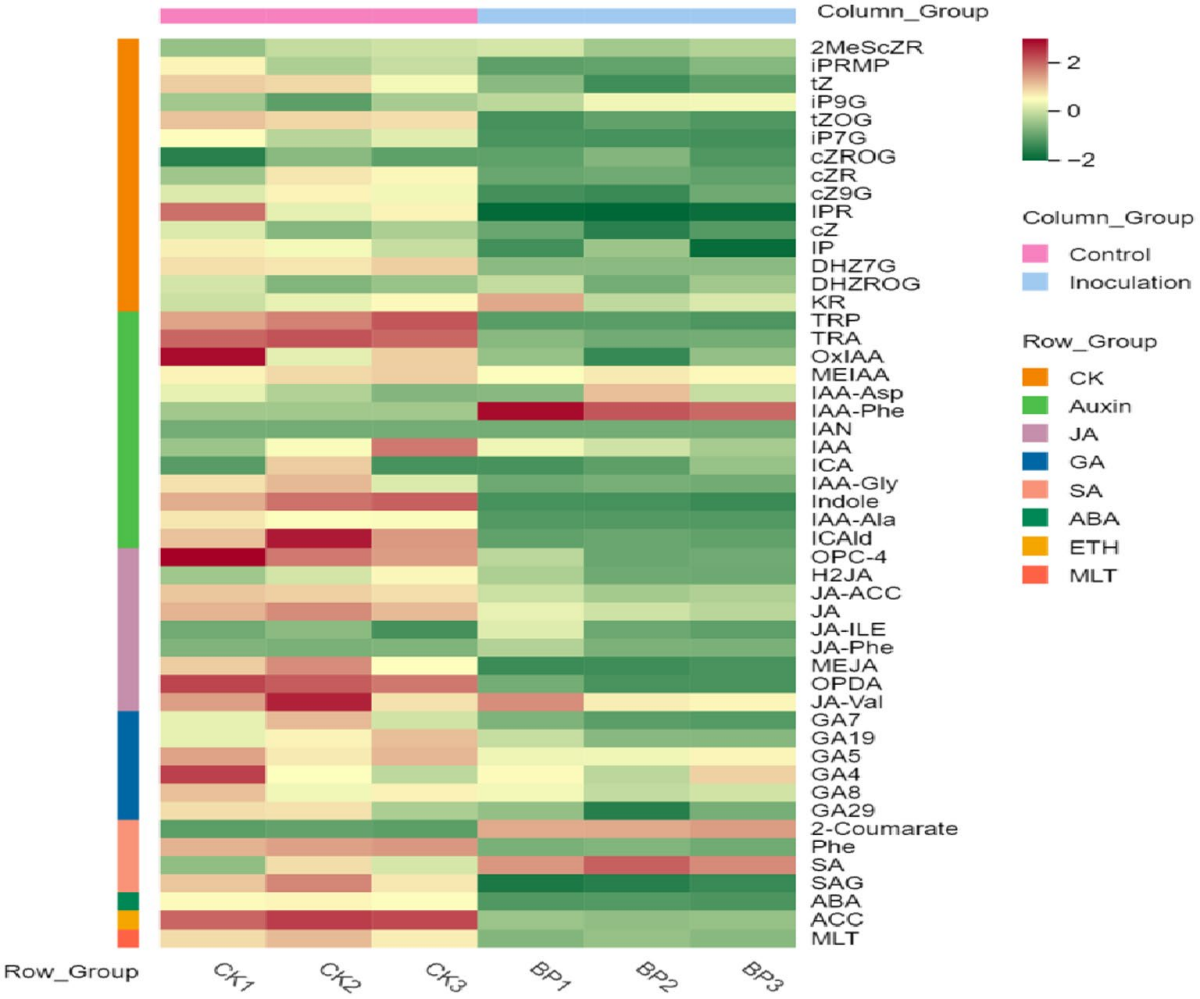
Sample population clustering diagram. Horizontal is the sample name, vertical is the metabolite information, and different colors are the colors filled with different values obtained after the standardized processing of different relative contents (red represents high content and green represents low content). The column group represents the group. The row class is the substance classification.

**Figure 6:**
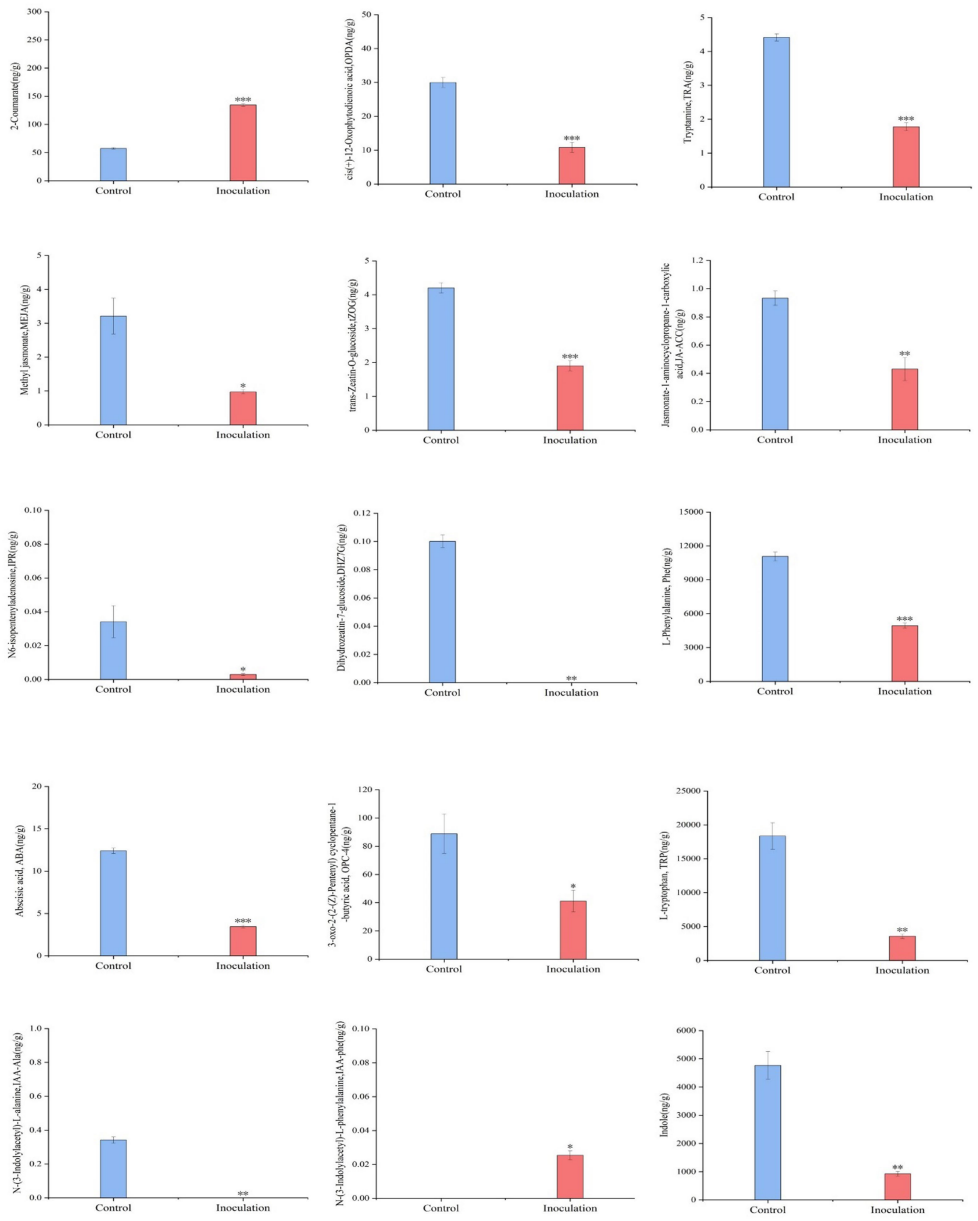
Histogram of the sample content. In the figure, the abscissa is the inoculated MXBP304 group and the uninoculated control group, and the ordinate is the hormone level. *p < 0.05, **p < 0.01, ***p < 0.001. *N* = 3.

First, the content data of plant hormones were processed using unit variance scaling (UV), and the heat map was drawn using the R software ComplexHeatmap package. Hierarchical cluster analysis (HCA) was performed to determine the accumulation patterns of metabolites in the different samples (Figure 5). Then, different hormones with fold change ≥2 and fold change ≤0.5 were selected by means of fold change analysis. The results showed that the expression of 15 plant hormones in the stems and leaves of *Digitaria sanguinalis* was significantly upregulated or downregulated after inoculation (Figure 6). We then used the KEGG database to annotate the metabolites, and the annotation results of the metabolite KEGG with significant differences were classified according to the KEGG pathway types, as shown in the following figure. The results showed that these 15 hormones were enriched in 24 KEGG pathways, mainly in metabolic processes, genetic information processing processes, and environmental information processing processes, among which the most enriched pathways were in metabolic processes (Supplementary Figure S1).

### 3.5 Gene expression in the stems and leaves of *Digitaria sanguinalis*

**Figure 7:**
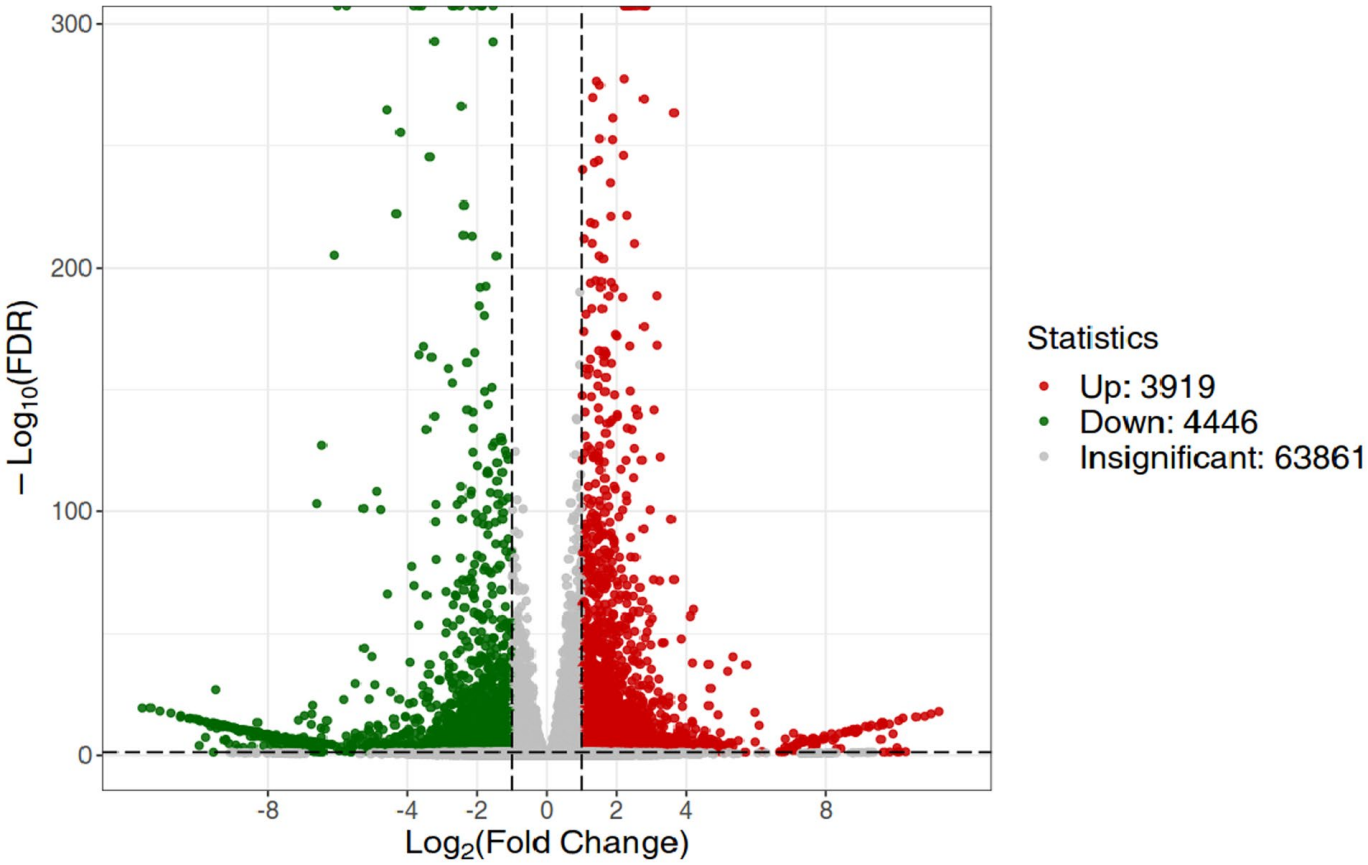
Differential gene volcano map. The horizontal coordinate indicates the change in gene expression, and the vertical coordinate indicates the significance level of the differential genes. Red dots represent differential genes with upregulated expression, green dots represent differential genes with downregulated expression, and gray dots represent non-differentially expressed genes.

**Figure 8:**
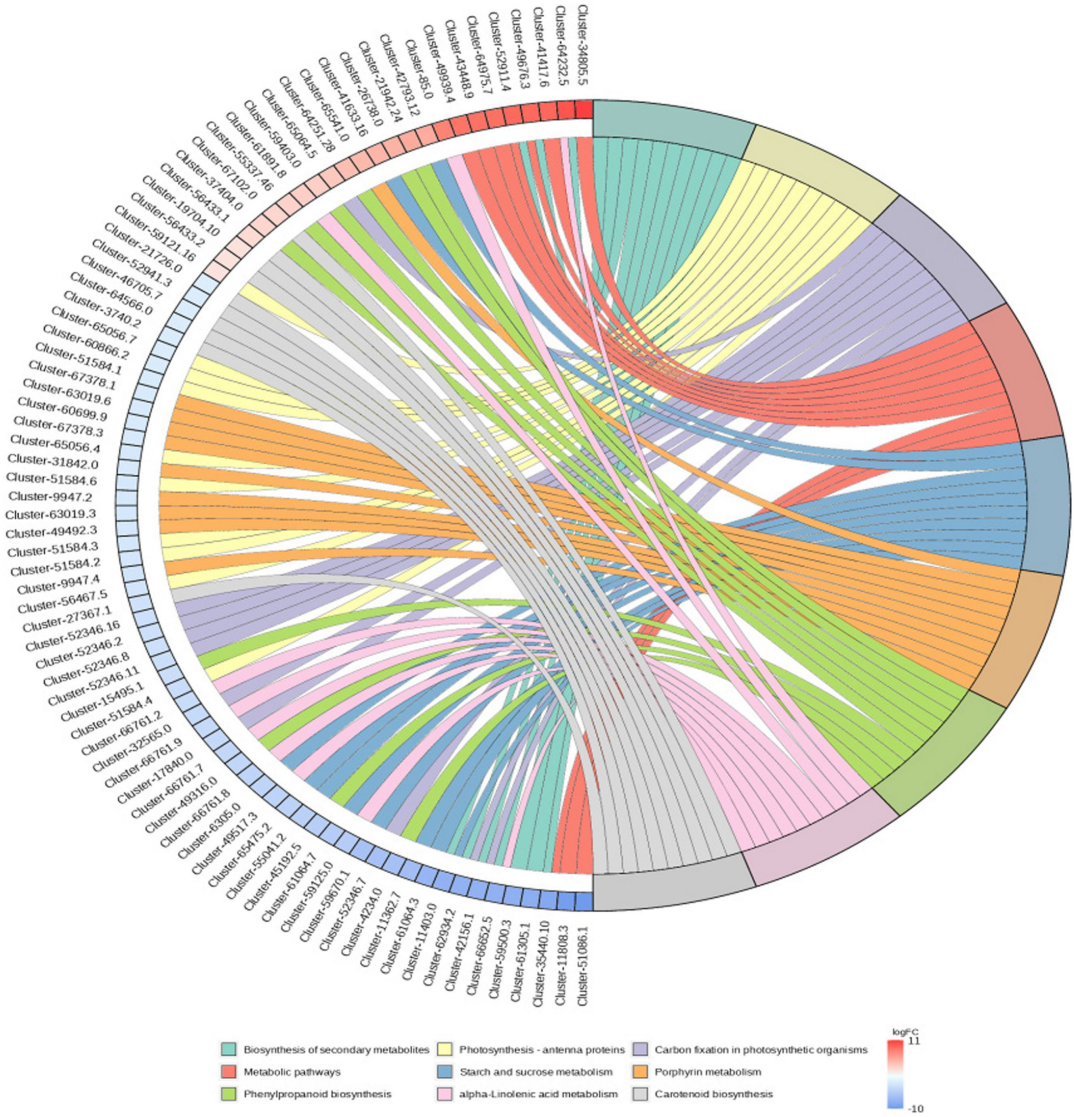
KEGG enriched chord diagram of *Mortierella*. The graph is divided into left and right sides: on the left are |logFC|, the top 10 genes in each classification; on the right are four metabolic pathways; the middle line represents the correspondence between pathways and genes; the lower right heat map shows the logFC value of genes; red is gene with upregulated expression, blue is gene with downregulated expression; the depth of the color indicates the size of logFC; the darker the color, the larger the multiple difference.

RNA-Seq data showed that there were 8,365 differentially expressed genes (DEGs) between the inoculation and control groups, among which the expression of 3,919 genes was upregulated and that of 4,446 genes was downregulated (Figure 7). After the genes were annotated in the KEGG database, a KEGG enrichment analysis of the DEGs was performed. We selected 50 KEGG pathways with the lowest q value in the enrichment analysis results and drew a histogram of the enrichment items. As shown in the figure below, the results of KEGG’s first classification level showed that differential genes were mainly enriched in five categories: metabolic process, genetic information processing process, environmental information processing process, cellular process, and organic system (Supplementary Figure S2). Next, we selected the nine channels with the lowest q value among the 50 channels and drew the enrichment analysis chord diagram. The results showed that the nine pathways with the lowest q values were derived from metabolic processes (Figure 8). In other words, after inoculation with *Mortierella* sp. MXBP304, the differential genes had the greatest influence on plant metabolic processes. The results have been uploaded to the NCBI database, accession number: PRJNA1082474.

### 3.6 Conjoint analysis

**Figure 9:**
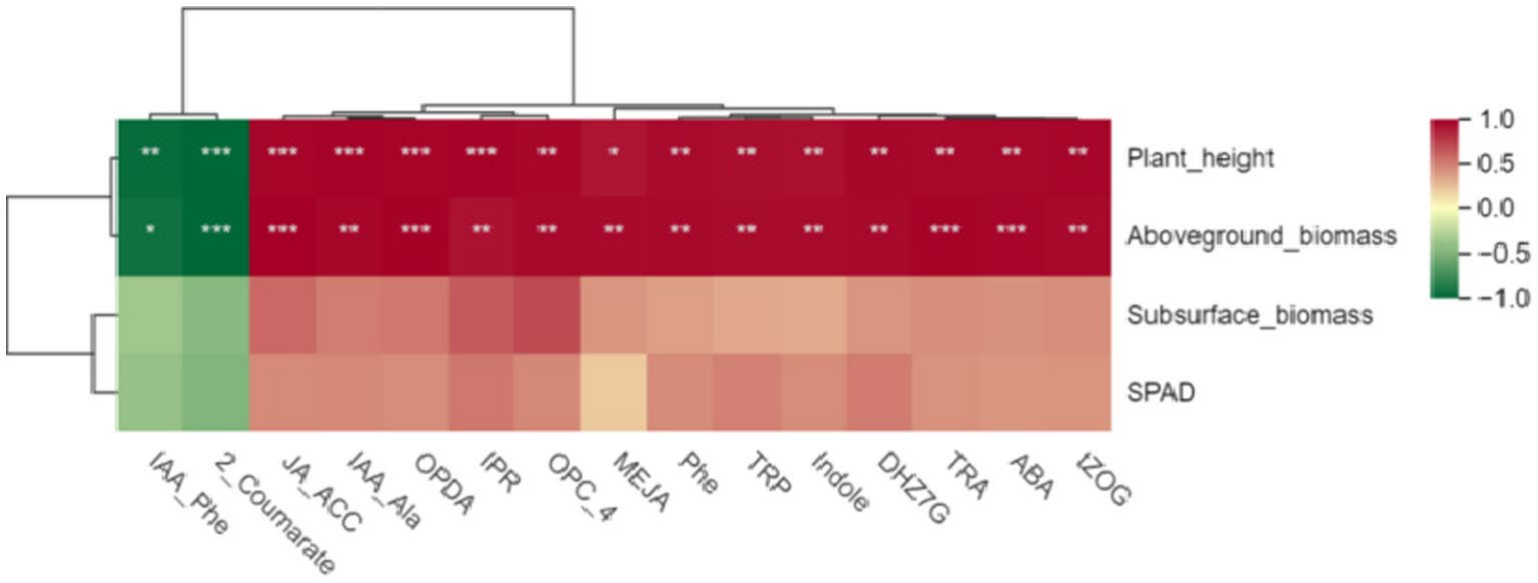
Heat map of different hormones and plant growth indices. The horizontal coordinate is the growth index of *Digitaria sanguinalis*, and the vertical coordinate is the differential hormone. SPAD: chlorophyll content; *p < 0.05, **p < 0.01, ***p < 0.001. ns indicates no significant difference. *N* = 3.

First, we conducted a pearson correlation analysis between these 15 hormones and the growth indicators of *Digitaria sanguinalis*. The results showed that there was no significant correlation between these 15 hormones and the chlorophyll and underground biomass of *Digitaria sanguinalis*. Among the 15 plant hormones, IAA-Phe and 2-Coumarate were negatively correlated with plant height and aboveground biomass, while the other 13 plant hormones were positively correlated with plant height and aboveground biomass (Figure 9). In other words, these 15 plant hormones were related to the plant height and biomass of *Digitaria sanguinalis*.

**Figure 10:**
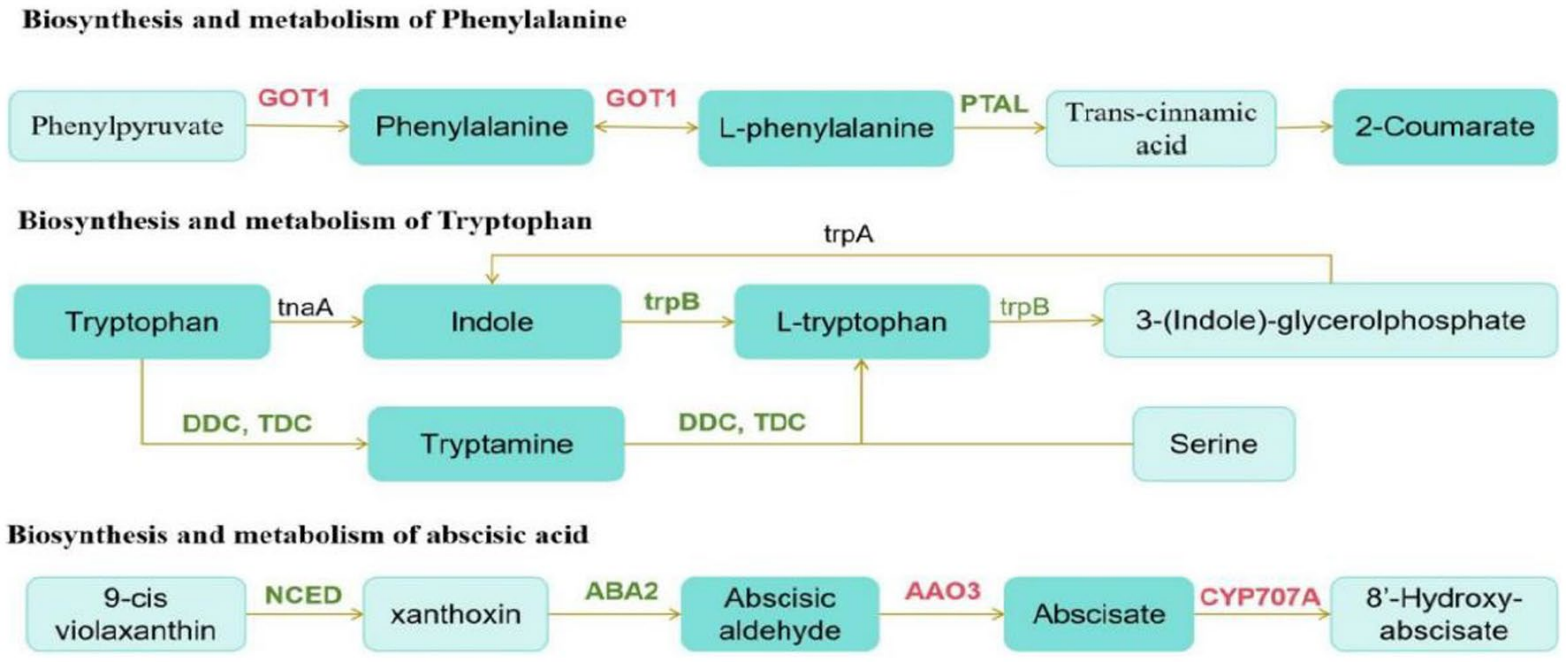
Schematic of metabolic pathways. The red text is the gene with upregulated expression, the green text is the gene with downregulated expression, and the black text is the non-differentially expressed gene. The dark green boxes indicate amino acid whose expression was significantly downregulated, and the light green boxes are substances that did not change significantly.

We then conducted a joint analysis of differential hormones and genes. First, we annotated *Digitaria sanguinalis* differential hormones and genes into the KEGG pathway at the same time and annotated 24 pathways. Among them, the top three pathways with the most annotated differential substances and differential genes were the metabolic, secondary metabolite synthesis, and plant hormone signal transduction pathways (Supplementary Figure S3). We conducted a comparative analysis of these 24 pathway maps and found that among these pathways, only genes directly related to the synthesis and metabolism of phenylalanine, tryptophan, and abscisic acid were differentially expressed after inoculation. Although DEGs and hormones were also noted in other pathways, they did not directly correspond. The synthesis and metabolic pathways of phenylalanine, tryptophan, and abscisic acid were reorganized and simplified (Figure 10).

According to the pathway map, the upregulation and downregulation in the expression of *GOT1* and *PTAL* directly affected phenylalanine synthesis and metabolism after inoculation. The downregulation in the expression of *trpB* and *DDC/TDC* genes in the tryptophan synthesis pathway significantly affects the synthesis and metabolism of indole and tryptophan. Phenylalanine and tryptophan are synthetic precursors of auxin. The decrease in these two substances further affects auxin synthesis. In the carotenoid biosynthesis pathway, the expression of the genes, *AAO3*, and *CYP707A*, which are directly related to the synthesis and metabolism of abscisic acid, is upregulated. Moreover, the *NCED* gene is also a key gene in the synthesis of abscisic acid. In this pathway, the downregulation in the expression of *NCED* significantly reduced the synthesis of abscisic acid.

## 4 Discussion

### 4.1 Response of *Mortierella* to the decline of growth indexes and plant hormones in *Digitaria sanguinalis*

In recent years, *Mortierella* species found in agricultural soils have come from different rhizosphere soils in different regions, such as wheat soil (Smit et al., 1999), and potato soil (Hýsek and Brožová, 2001). In maize (Li et al., 2018), ginseng (Bian et al., 2020), and sorghum (Silva et al., 2017) rhizosphere soils. To the best of our knowledge, *Mortierella* has not been isolated from the rhizosphere soil of alfalfa, a newly discovered *Mortierella* species. In addition, many studies have found that the application of Sporoides has positive effects on soil activity and plant growth parameters (including crop species). For example, Sporoides in salt-invaded coastal soils in China can increase plant roots and aboveground dry weight. Furthermore, the activities of urease, neutral phosphatase, alkaline phosphatase, and catalase in the soil increased (Zhang et al., 2011). *Mortierella* isolated from maize rhizosphere soil can also increase the dry weight of plants, the concentrations of IAA and ABA in roots, and the concentrations of available nitrogen and phosphorus in soil to varying degrees (Li et al., 2020). Sporoides in the soil of saffron planted in India not only increased plant biomass but also chlorophyll and carotenoid content (Wani et al., 2017), but high-throughput sequencing results showed that *Mortierella* is the dominant pathogen of soybean varieties HN66 and HN48 (Guo et al., 2021). Therefore, the growth-promoting effects of *Mortierella* may be plant-specific. Our results showed that the newly discovered strain *Mortierella* sp. MXBP304 had a significant inhibitory effect on weed *Digitaria sanguinalis* biomass, plant height, and plant hormones ABA, IAA, and CK, which differed from the promoting effect of *Mortierella* reported in previous studies. Based on previous studies, we speculated that the reason for this inhibitory effect may be the difference between the species of *Mortierella* and the host plant.

### 4.2 The key genes involved in the synthesis and metabolism of abscisic acid, phenylalanine, and tryptophan were regulated by *Mortierella* sp. MXBP304

During plant growth, plant hormone signaling pathways are activated when the environment stimulates plants, thus playing an important role in responding to various biological and abiotic stresses (Egamberdieva, 2013). In addition, transcription factors play an important role in plant resistance to various environmental stresses (Wang et al., 2006). The application of exogenous microbial inoculants can cause changes in the plant environment, which is equivalent to biological stress. As an important plant hormone, ABA plays an important role in plant responses to biological and abiotic stressors (Li and Liu, 2019). Some studies have reported that the association between fungi and plants regulates the expression of ABA-related genes (Porcel et al., 2012). *NCED* is a key enzyme in ABA synthesis (Lin et al., 2007). Some studies have shown that ABA biosynthesis genes *ZEP* and *NCED* increase ABA levels in seeds (Tuan et al., 2018). However, the ABA catabolic *CYP707A* gene family reduces ABA levels (Frey et al., 2012). In this study, the *NCED* and *CYP707A* gene families were detected in *Digitaria sanguinalis* plants after inoculation with the exogenous microbial inoculant strain MXBP304. Among them, the expression of *CYP707A* is upregulated, expression of *NCED* is downregulated, and ABA level decreased, which is consistent with the results of previous studies (Kushiro et al., 2004).

The phenylpropane metabolic pathway is an important metabolic pathway in plant growth, development, and metabolism, including the phenylalanine, coumaric acid, and flavonoid pathways (Muro-Villanueva et al., 2019). The phenylalanine pathway is the core pathway of phenylpropane metabolism, which starts with phenylalanine, passes through phenyl pyruvic acid, coumaric acid, and other intermediate products, and finally synthesizes a variety of bioactive secondary metabolites, such as flavonoids, tannins, anthocyanins, and vanillic acid (Craven-Bartle et al., 2013). In this study, the *GOT1* and *PTAL* genes, which directly regulate the synthesis and metabolism of phenylalanine and L-phenylalanine in the phenylpropane metabolic pathway, underwent significant changes. Studies have shown that *PTAL* can catalyze the deamination of phenylalanine and tyrosine simultaneously in the phenylpropane pathway and participate in the synthesis of downstream secondary metabolites, such as lignin and flavonoids (Barros et al., 2016). *GOT* plays an important role in regulating nitrogen metabolism and has a significant impact on plant quality and yield (Reiner et al., 2010).

Tryptophan is an important amino acid in plants, a physiological prerequisite for plant growth hormone (IAA) synthesis, and the metabolic precursor of many secondary metabolites (Samani et al., 2019). Tryptophan synthase (*LetrpB*) is the key rate-limiting enzyme in the final step of tryptophan synthesis. Overexpression of tryptophan synthase can also increase the number of Arabidopsis side roots. Simultaneously, biomass increased to a certain extent (Sanjaya et al., 2008). In this study, the expression of *trpB* was downregulated, and the level of tryptophan was reduced, which is consistent with previous studies.

### 4.3 The *Mortierella* sp. MXBP304 inhibited the growth of *Digitaria sanguinalis* directly by affecting plant hormone content

Tillering, an important component of plant type in the grass family, plays an important role in crop yield and quality. The regulatory effect of ABA on lateral branches is related to the promotion of lateral bud dormancy and the inhibition of lateral bud germination and growth. It has been reported that an increase in ABA levels during tillering of rice and wheat inhibits tillering and its subsequent growth and development (Liu et al., 2011; Dong et al., 2023). In other words, the increase in ABA levels inhibits plant growth, and the decrease in ABA levels in this experiment suggests that ABA is not the key hormone that inhibits the growth of *Digitaria sanguinalis*. It has been reported that phenylalanine can significantly improve the carbon and nitrogen content and photosynthetic production capacity of wheat seedlings (Nguyen et al., 2016). L-phenylalanine is a precursor of phenylpropanoid metabolism in plants and an aromatic amino acid, which has an impact on many physiological processes, including plant development and growth, signal transmission, metabolizing energy production, and plant resistance to stress (Hildebrandt et al., 2015; Teixeira et al., 2017). Some studies have also found that spraying L-phenylalanine on a sage leaf surface can increase the fresh and dry weight of sage and promote its growth (Samani et al., 2019). L-phenylalanine can also significantly improve the activity of nitrate reductase and catalase in maize and promote the absorption of nitrogen, phosphorus, potassium and zinc by maize (Chen et al., 2005). It can also significantly enhance the activity of superoxide dismutase, peroxidase, and phenylalanine ammoniase in thyme adventite buds (Li X. D. et al., 2016). Studies have shown that tryptophan can promote the growth of wheat, improve its antioxidant activity and photosynthetic characteristics (Qiang, 2019). Thus, phenylalanine and tryptophan have important effects on plant growth and development. In this study, regulating the differential gene expression of phenylalanine and tryptophan reduced the content of these two amino acids, so it is inferred that the decrease in the biomass of *Digitaria sanguinalis* is related to phenylalanine and tryptophan to a certain extent.

## 5 Conclusion

After inoculation with *Mortierella* sp. MXBP304, the plant height and aboveground biomass of *Digitaria sanguinalis* decreased significantly, and the related enzyme activities of *Digitaria sanguinalis* also changed to different degrees. During the growth of *Digitaria sanguinalis*, the expression of *GOT1* and *PTAL* genes was upregulated and downregulated in the phenylalanine synthesis and metabolism pathways, respectively, directly affecting the relative content of phenylalanine. Downregulation of the expression of *trpB* and *DDC/TDC* genes significantly affected the relative contents of indole and tryptophan in the tryptophan synthesis pathway. Therefore, through a comprehensive analysis of physiological indexes, plant hormone indexes, and transcription factors of *Digitaria sanguinalis*, this study proposed the hypothesis mechanism of inhibiting the effect of *Mortierella* sp. MXBP304 on *Digitaria sanguinalis*. Inoculation significantly affected the upregulation and downregulation in the expression of *GOT1* and *PTAL* genes in the phenylalanine biosynthesis and metabolism pathways and the downregulation in the expression of *trpB* and *DDC/TDC* genes in the tryptophan synthesis pathway. The upregulation and downregulation in the expression of these genes significantly reduced the relative levels of phenylalanine and tryptophan. The decrease in phenylalanine and tryptophan levels directly inhibited the biomass and plant height of *Digitaria sanguinalis*.

## Data availability statement

The datasets presented in this study can be found in online repositories. The names of the repository/repositories and accession number(s) can be found in the article/Supplementary material.

## Author contributions

TD: Data curation, Methodology, Project administration, Writing – original draft. XDQ: Formal analysis, Investigation, Writing – review & editing. YW: Project administration, Software, Writing – review & editing. ML: Conceptualization, Investigation, Writing – review & editing. XHQ: Investigation, Supervision, Writing – review & editing. JJ: Conceptualization, Investigation, Methodology, Writing – review & editing. YG: Investigation, Validation, Writing – review & editing. ZW: Conceptualization, Methodology, Resources, Writing – review & editing. KL: Resources, Supervision, Writing – review & editing. CY: Funding acquisition, Methodology, Supervision, Writing – review & editing. JS: Funding acquisition, Methodology, Project administration, Supervision, Writing – review & editing.

## Footnotes

^1^http://www.kegg.jp/kegg/compound/

^2^http://www.kegg.jp/kegg/pathway.html

^3^http://www.genome.jp/kegg/
