# Supplementary material for: Rhizosphere Mortierella strain of alfalfa exerted weed growth inhibition by inducing expression of plant hormone-related genes
Source: Front Microbiol. 2024 Jun 17;15:1385992. doi: 10.3389/fmicb.2024.1385992 (PMC11215053; doi:10.3389/fmicb.2024.1385992)
Supplement: Supplementary file 1 [file Data_Sheet_1.docx]

**Supplementary Material**

## Supplementary Figures


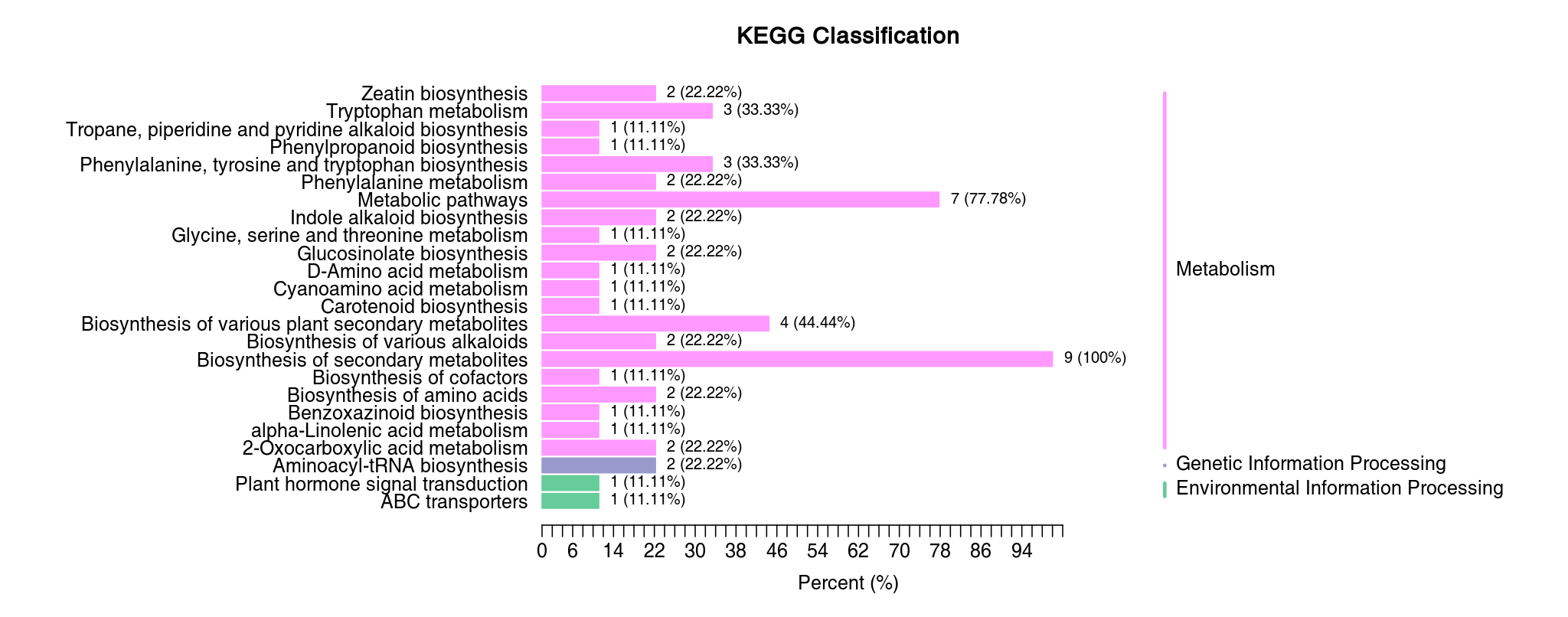


**Fig. S1** KEGG classification of differential metabolites.The ordinate is the name of the KEGG metabolic pathway, the number in the figure indicates the number of differential metabolites annotated to this pathway, and the ratio of the number of differential metabolites annotated to this pathway to all the differential metabolites annotated to KEGG is in parentheses.


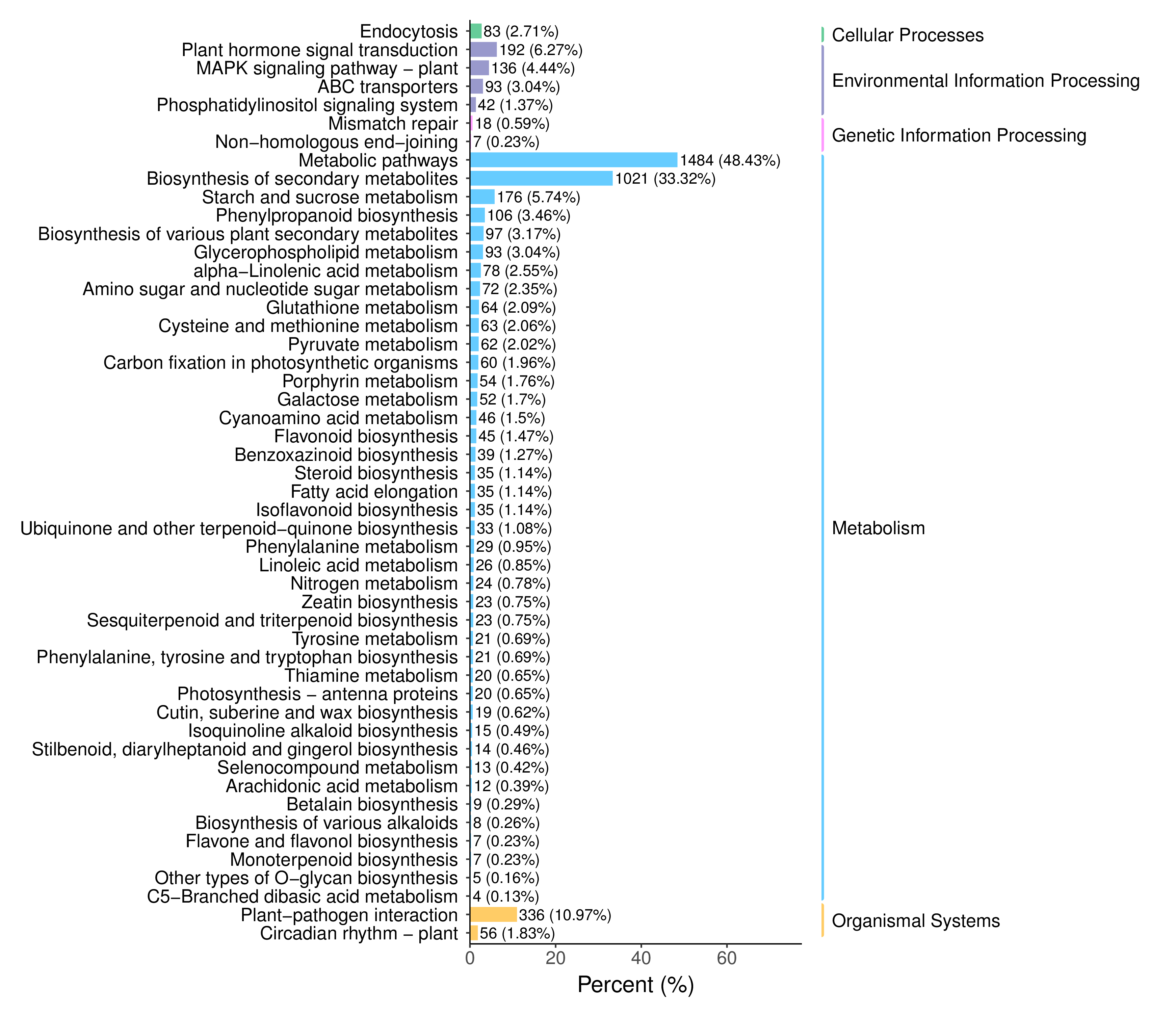


**Fig. S2** KEGG enrichment column of differential gene.The horizontal coordinate indicates the number of differential genes annotated to the pathway, and the vertical coordinate indicates the name of the KEGG pathway. Numbers in the figure represent the number of differential genes annotated to this pathway, parentheses are the ratio of the number of differential genes annotated to the number of annotated differential genes, and the label on the far right represents the classification to which the KEGG pathway belongs.


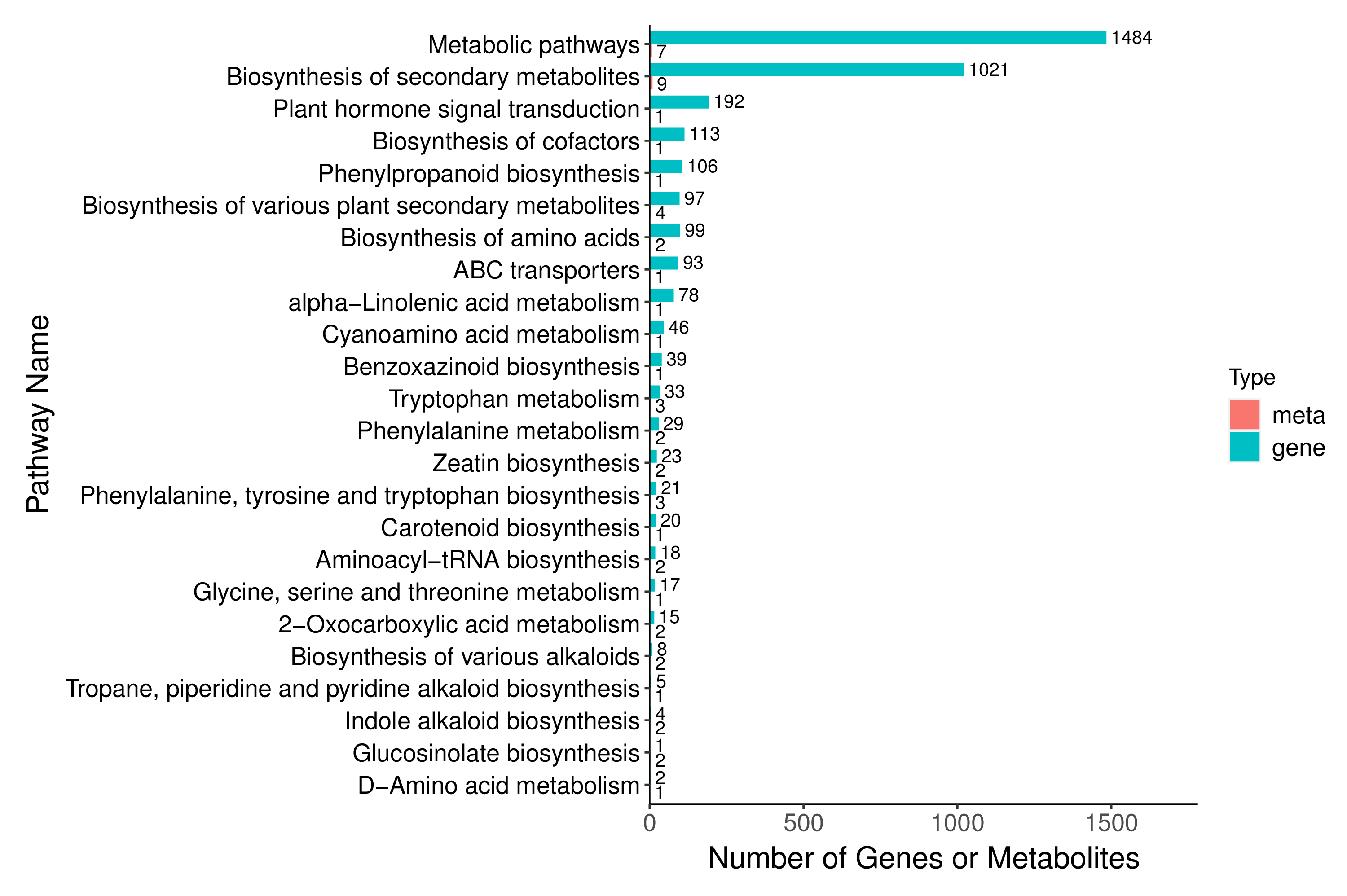


**Fig. S3** Bar chart of KEGG enrichment analysis.The horizontal coordinate represents the number of differential metabolites and differential genes enriched into this pathway, the vertical coordinate represents the KEGG pathway name, and the red and green bars represent the metabolome and transcriptome respectively.
